# On the Structural and Chemical Characteristics of Co/Al_2_O_3_/graphene Interfaces for Graphene Spintronic Devices

**DOI:** 10.1038/srep14332

**Published:** 2015-09-23

**Authors:** Bárbara Canto, Cristol P. Gouvea, Bráulio S. Archanjo, João E. Schmidt, Daniel L. Baptista

**Affiliations:** 1 Instituto de Física, PPGFis, PPGMicro, Universidade Federal do Rio Grande do Sul, Porto Alegre, 91501-970, RS, Brazil; 2 Divisão de Metrologia de Materiais, INMETRO, Duque de Caxias, 25250-020, RJ, Brazil

## Abstract

We report a detailed investigation of the structural and chemical characteristics of thin evaporated Al_2_O_3_ tunnel barriers of variable thickness grown onto single-layer graphene sheets. Advanced electron microscopy and spectrum-imaging techniques were used to investigate the Co/Al_2_O_3_/graphene/SiO_2_ interfaces. Direct observation of pinhole contacts was achieved using FIB cross-sectional lamellas. Spatially resolved EDX spectrum profiles confirmed the presence of direct point contacts between the Co layer and the graphene. The high surface diffusion properties of graphene led to cluster-like Al_2_O_3_ film growth, limiting the minimal possible thickness for complete barrier coverage onto graphene surfaces using standard Al evaporation methods. The results indicate a minimum thickness of nominally 3 nm Al_2_O_3_, resulting in a 0.6 nm rms rough film with a maximum thickness reaching 5 nm.

Graphene is a potential material for spintronic applications because of the combination of its expected long spin lifetime and high electron mobility. The spin diffusion distances observed in graphene are very long; i.e., few micrometers at room temperature^1^,^2^,^3^,^4^,^5^,^6^. Experimentally, graphene spin-injection devices can be obtained by fabricating ferromagnetic metal contacts on graphene; these assemblies act as spin-current filters. However, previous studies showed that electrical spin injection from such ferromagnetic electrodes in direct contact (transparent contact) with graphene is not effective because of the conductance mismatch^1^,^7^. Instead, the use of a thin insulating layer acting as a tunnel barrier (tunneling contact) between the graphene layer and the metal electrodes has proven to be an effective solution^3^,^4^,^8^,^9^,^10^. Han *et al.* observed an increase of the injection efficiency from 1 to 26–30% by using tunneling contacts. Concomitantly, the spin relaxation time was also enhanced by more than ninefold, reaching 771 ps at room temperature^4^. The effect of direct metal contacts on spin lifetime measurements in graphene was investigated by Maassen *et al.*^11^ An important discussion on the effect of low resistance contact-induced spin relaxation on Hanle precession curves is afforded. Recently, a theoretical closed-form expression for Hanle spin precession in different regimes was also provided, clearly demonstrating the influence of metal contacts on the spin relaxation mechanisms and also the importance of using tunneling contacts^12^. Nevertheless, complete control of standard tunneling barrier fabrication on graphene sheets is still distant^1^,^4^,^13^,^14^,^15^,^16^,^17^,^18^. Barrier structural and chemical non-uniformities seem to play a crucial role in the experimental spin relaxation time values; these are much shorter than expected (*ca.* microsecond)^1^,^3^,^4^,^19^ from the low intrinsic spin-orbit couplings of graphene^20^. Barrier pinholes (pinhole contacts) are one of the barrier defects that may lower the metal/barrier/graphene interface quality, directly affecting spin injection and relaxation through the graphene^1^,^3^,^19^.

Tombros *et al.*^1^ noted that evaporated alumina (aluminum evaporation followed by oxidation) is commonly used to construct tunneling barriers. However, the possible existence of pinholes remains an important issue for the development of standard fabrication procedures for 1–5-nm-thick barriers^1^,^13^. Han *et al.* also evaluated the influence of barrier roughness on the spin relaxation mechanisms by using molecular beam epitaxy MgO and TiO_2_ seed layers as an alternative for the fabrication of smoother barrier layers on graphene^14^. Dublak *et al.* demonstrated the use of sputtering to deposit continuous 1-nm-thick Al_2_O_3_ onto graphene, but this technique damaged the graphene structure and hence reduces the applicability of such an approach^15^,^16^. Fluorinated-graphene^21^ and h-BN^22^,^23^ monolayers were also successfully used as tunneling barriers; however, sensitive chemical processes and/or critical layer transfer steps are added. One such alternative approach enhanced important spin transport metrics, but the results have still not reached much longer spin relaxation times as expected^20^. Recently, hydrogenated-graphene barrier was also proposed; the lower spin polarization was justified by the authors due to the possible presence of magnetic moments acting as spin scatterers in such tunnel barrier^24^. Thus, a complete control and understanding of the structural and chemical nature of tunneling barriers on graphene and the role of graphene and barrier defects on spin injection and relaxation is still an experimental challenge. A detailed investigation concerning the metal/barrier/graphene interface nature using direct electron microscopy visualization and nanometer-resolved spectrum profiles is currently absent from the literature.

In this work, we report a detailed investigation of the structural and chemical characteristics of traditional thin evaporated Al_2_O_3_ barriers with variable thickness grown onto single-layer graphene sheets. Advanced electron microscopy and spectrum-imaging techniques were used to investigate the Co/Al_2_O_3_/graphene/SiO_2_ and Co/graphene/SiO_2_ interfaces. A direct cross-sectional observation of barrier pinholes is reported as well as results concerning the minimal barrier thickness necessary for complete graphene coverage using standard Al evaporation. The results are compared with standard thin Al_2_O_3_ deposition onto SiO_2_/Si substrates.

## Results and Discussions

**Figure 1:**
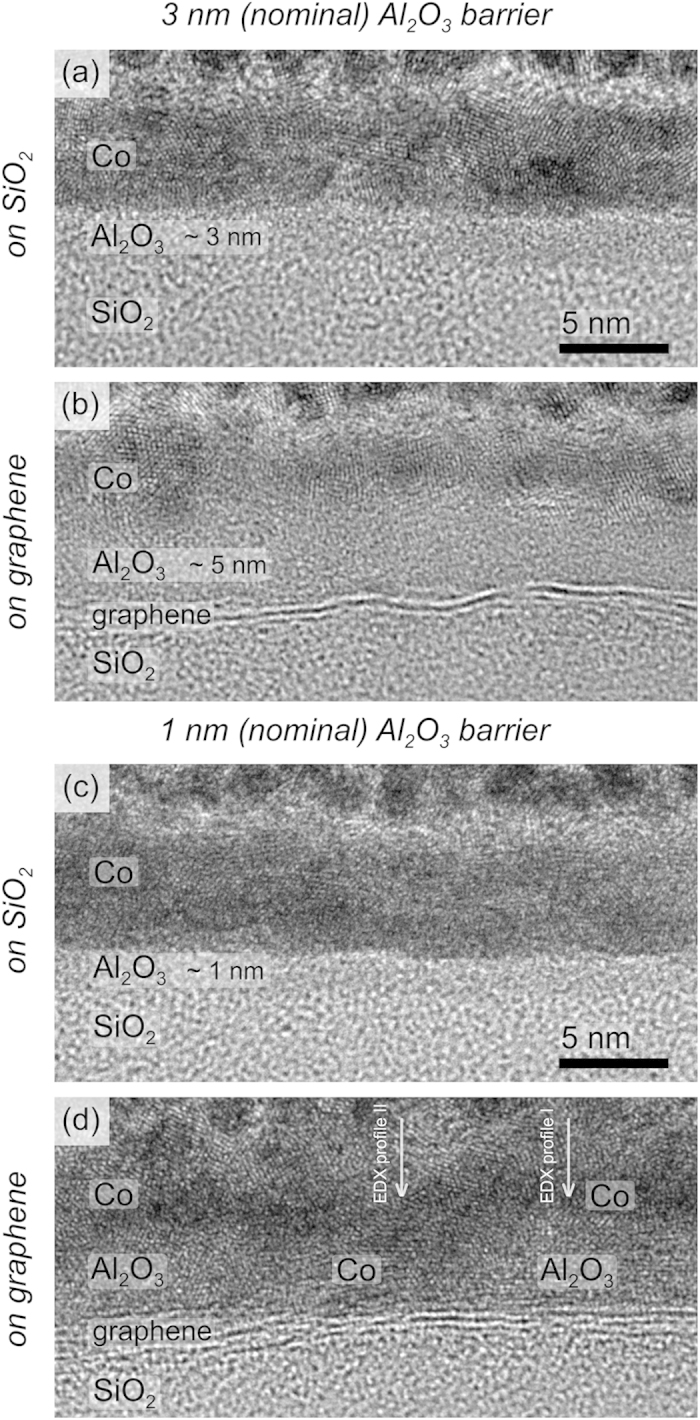
TEM cross-sectional images of samples with different Al_2_O_3_ barrier thicknesses, 3-nm-thick (nominal) barrier on (**a**) SiO_2_ and (**b**) graphene, 1-nm-thick (nominal) barrier on (**c**) SiO_2_ and (**d**) graphene.

**Figure 2:**
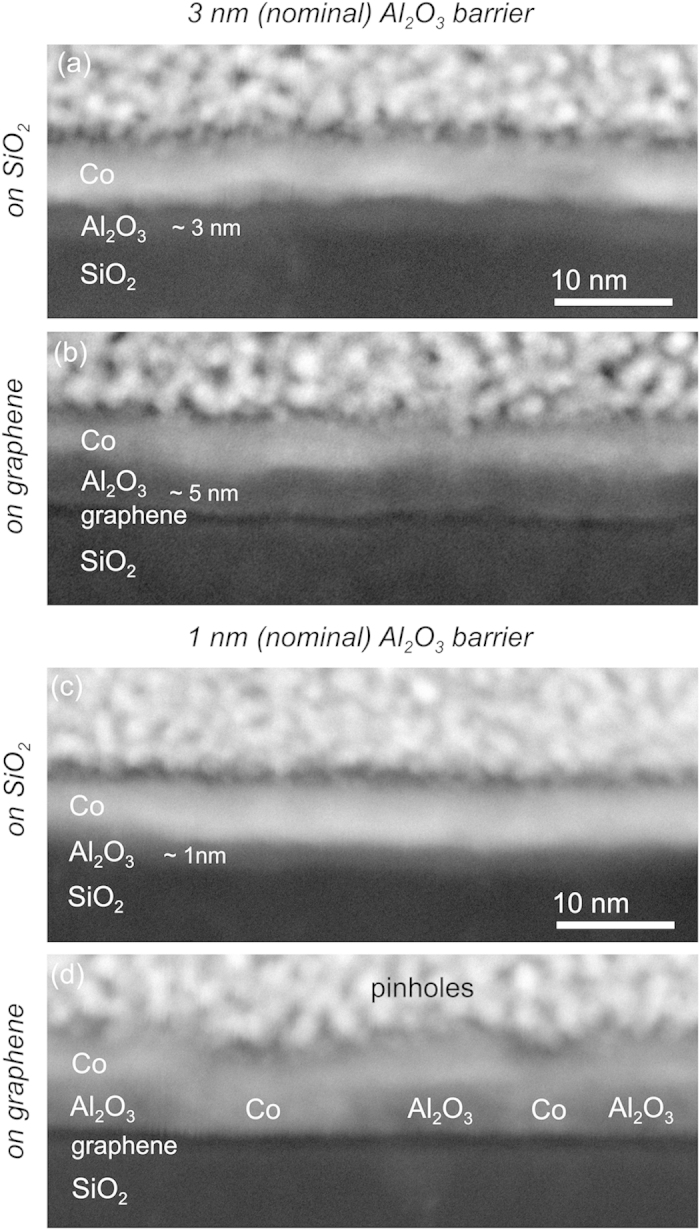
HAADF-STEM cross-sectional images of samples with different Al_2_O_3_ barrier thicknesses, 3-nm-thick (nominal) barrier on (**a**) SiO_2_ and (**b**) graphene, 1-nm-thick (nominal) barrier on (**c**) SiO_2_ and (**d**) graphene.

The Co-coated evaporated Al_2_O_3_ barrier samples were analyzed systematically by advanced (S)-TEM techniques. Figures 1 and 2 show TEM and STEM cross-sectional images, respectively, of samples with different Al_2_O_3_ barrier thicknesses. The analyses included two substrate regions: with and without graphene. It is important to notice that each Al_2_O_3_ barrier sample presents regions with and without graphene at the same substrate, leading to the same nominal barrier deposition for both graphene/SiO_2_ and SiO_2_ regions. Parts (a) and (c) of both Fig. 1 (TEM) and 2 (STEM) correspond to images of the 3- and 1-nm-thick barriers deposited directly onto the SiO_2_ surface in a region without graphene. For both barrier thicknesses, it is possible to observe a homogenous layer covering the whole substrate surface. The Co layer is also observed as well as the top granular platinum-capping region, purposely deposited during the FIB lamella preparation to protect the integrity of the sample interface layers. It is important to note that both TEM and STEM images present similar features but in reverse contrast. The HAADF STEM images (Fig. 1) contain a high-Z contrast dependence where high-Z regions are imaged through the annular detector as brighter zones. In such cases, the Co layer is imaged brighter than the Al_2_O_3_ one.

A different morphological behavior was observed when the Al_2_O_3_ was deposited onto the graphene sheets. Figures 1(b,d) and 2(b,d) show cross-sectional images of monolayer graphene samples with nominally 3- and 1-nm-thick Al_2_O_3_ barriers. The images clearly show that the barrier thickness is larger compared with the same barrier deposited directly onto the SiO_2_. The maximum thickness reached *ca.* 4 and 5 nm for respectively the nominally 1- and 3-nm-thick Al_2_O_3_ layers. Both TEM and STEM analyses showed that the surface coverage was incomplete: a few nanometers-large pinholes are evident in the graphene samples coated with the nominally 1-nm-thick Al_2_O_3_ barrier. The pinholes are especially clear in the STEM images, where the Co filling inside the holes is also visible. As noted above, the Co appears brighter than the Al_2_O_3_ in HAADF STEM images. Thus, the Co layer clearly contacts the graphene directly in those pinhole regions.

In contrast, the graphene sample with a nominally 3-nm-thick Al_2_O_3_ layer did not contain pinholes. An Al_2_O_3_ layer having a maximum thickness of *ca.* 5 nm and completely covering the whole graphene surface was observed.

**Figure 3:**
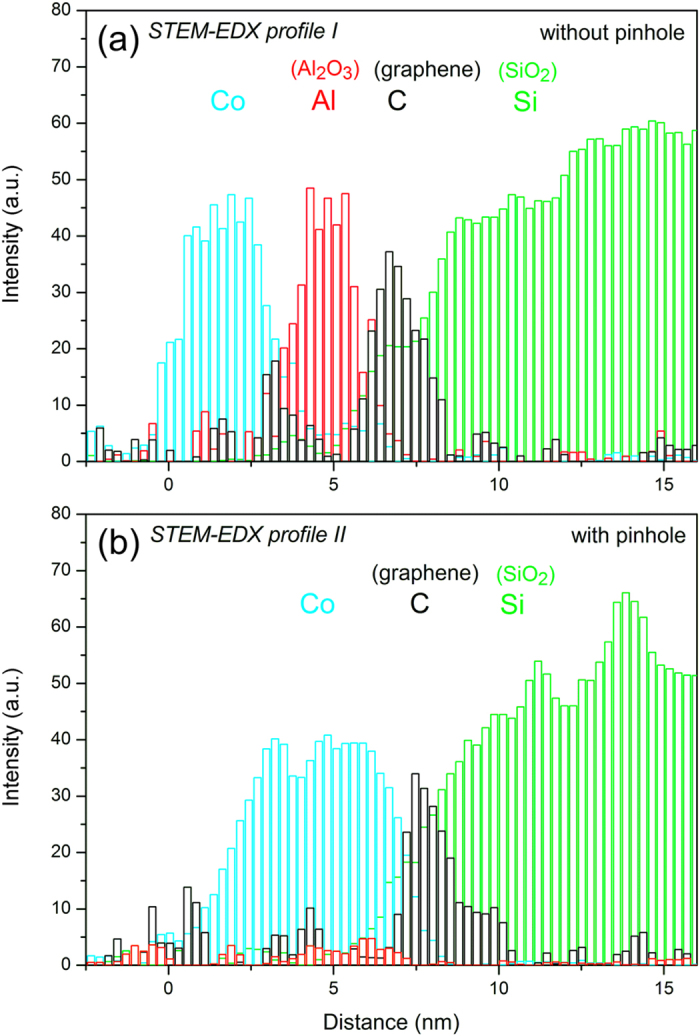
Normalized-intensity EDX profile along a section (**a**) without pinhole and (**b**) with pinhole for the nominally 1-nm-thick barrier sample.

The chemical nature of the Co/Al_2_O_3_/graphene/SiO_2_ interfaces at different regions was also probed by means of STEM–EDX spectrum profiling experiments. Figure 3(a) shows the normalized-intensity EDX profile along a section without pinhole for the nominally 1-nm-thick barrier sample (indicated by the arrow in Fig. 1(d)). As expected, the profile confirmed the presence of a well-defined Al_2_O_3_ layer between the graphene and the Co film. The thicknesses of the layers are consistent with those obtained by TEM and STEM although they appear larger because of the lower spatial resolution of the EDX technique. When the same profiling experiment was acquired for a region with pinholes (indicated by the arrow in Fig. 1(d)), the EDX Al signal from the Al_2_O_3_ barrier disappeared, confirming the formation of a direct interface between the graphene and the Co layer (Fig. 3(b)).

Such non-homogeneity of the Al_2_O_3_ barrier on the graphene is attributed to agglomeration during the Al deposition. The low-energy graphene surface induces high diffusion mobility of atomic species during Al deposition, which leads to cluster-like film (Volmer-Weber) growth^25^. The coverage on graphene can be partial and the barrier thickness is greater than nominal. This clustering phenomenon limits the minimal thickness possible for complete barrier coverage on graphene surfaces using standard Al evaporation methods. In such case, the results indicate an Al_2_O_3_ layer having a minimum thickness of nominally 3 nm, which means thickness reaching a maximum thickness of *ca.* 5 nm. Although the nominal (3 nm) thickness may lead to complete barrier coverage, AFM measurements indicated a relative high rms roughness of ~0.6 nm (see Supplementary Fig. S2 online).

**Figure 4:**
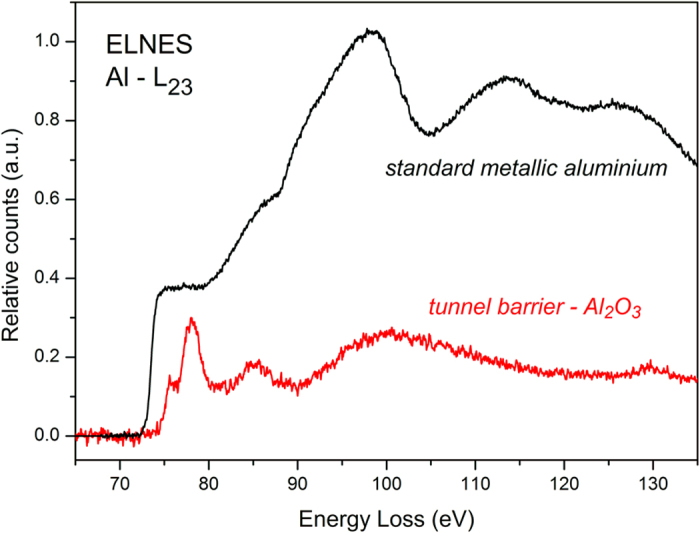
Spatially resolved EELS of the tunnel barrier showing the Al L-edge spectrum, the near-edge fine structure is consistent with the presence of Al from an Al_2_O_3_ phase, indicating complete oxidation of the evaporated Al layer; a spectrum of a standard metallic Al sample is also showed for comparison.

The chemical nature of the barrier was also investigated using spatially resolved EELS during STEM–HAADF experiments. Figure 4 shows the Al L-edge spectrum of the 3-nm-thick (nominal) barrier sample. The analysis of the near-edge fine structure is consistent with the presence of Al from an Al_2_O_3_ phase, indicating complete oxidation of the evaporated Al^26^,^27^.

**Figure 5:**
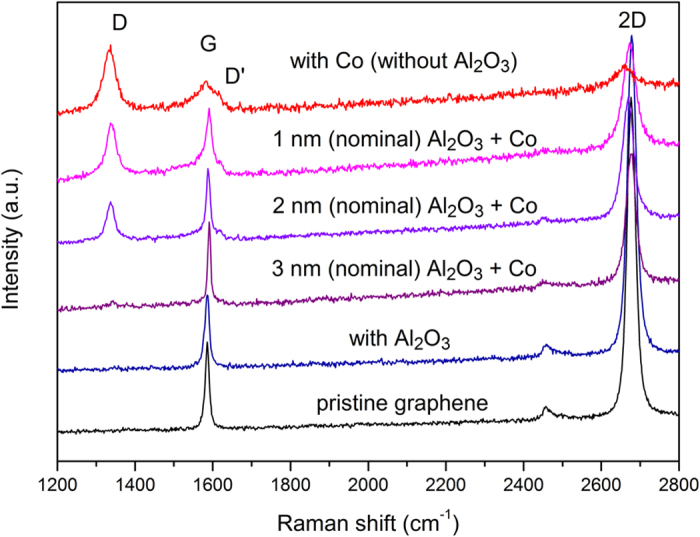
Raman spectra of graphene at each fabrication step, the pristine exfoliated graphene presents a typical spectrum for a high quality structure with absent D peak and high 2D one, the effect of the Al_2_O_3_ barrier and the Co deposition on the graphene atomic structure quality is showed.

Raman spectroscopy was used to probe the effect of the Al_2_O_3_ barrier and the Co deposition on the graphene atomic structure quality; Fig. 5 shows the spectra acquired at each fabrication step. No apparent damage was observed for any barrier thickness. The diffusive character of the thermal evaporation of aluminum leads to non-energetic species deposition, which preserves the graphene integrity. Similar results have been reported in the literature for other tunnel barriers thermally deposited onto graphene sheets.

The use of Co sputtering deposition during the fabrication of ferromagnetic contacts may lead to moderate graphene damage. The Raman spectrum of graphene after direct (without barrier) Co deposition contained a prominent defects-related D band^28^. This can be explained by momentum transference among energetic (a few eV) Co species and carbon atoms in graphene through knock-on collisions at the very beginning of the Co film deposition. The use of tunnel barriers between the graphene and the Co contacts may prevent such damage. Stopping and range of ions in matter (SRIM) simulations^29^ (not shown) indicated that even very thin (1 nm) Al_2_O_3_ barriers are very thick for few-eV sputtered Co species, and are sufficiently thick to prevent any damage to the graphene. However, the presence of pinholes in the barrier structure provides a direct path for Co–carbon collisions. Figure 5 shows the Raman spectra of graphenes after Co deposition having 1-, 2- and 3-nm-thick (nominal) Al_2_O_3_ barriers. The presence of the D band for the samples with the 1- and 2-nm-thick barriers clearly indicates the presence of inhomogeneous barriers containing many pinholes (see Supplementary Fig. S3 online). It is important to note that the I_D_/I_G_ ratio is higher for the sample without a barrier, and that this ratio decreases as the thickness of the barrier increases. This indicates progressive coverage of the surface by the Al_2_O_3_ barrier. The D band is absent for the 3-nm-thick (nominal) barrier samples, confirming that this thickness is the approximate limit for complete coverage of the graphene by the Al_2_O_3_. The Raman analyses are in concordance with the electron microscopy results, indicating that Raman spectroscopy in such experiment is a useful technique for probing the existence of pinholes in tunnel barriers on graphene over large areas.

In conclusion, direct observation of pinhole contacts was achieved using FIB cross-sectional and advanced high-resolution TEM and STEM analyses. Spatially resolved EDX spectrum profiling showed the nature of direct point-contacts between the Co ferromagnetic layer and the graphene. Raman spectroscopy indicated that moderate damage occurred over large areas of the graphene during the Co sputtering deposition. The presence of pinholes in the barrier structure provided a direct path for Co-carbon collisions. Such pinholes were widely distributed on the samples having 1- and 2-nm-thick (nominal) Al_2_O_3_ barriers. Only the 3-nm-thick (nominal) barrier provided complete coverage of the graphene surface, thereby preserving the graphene integrity during the Co deposition. No pinholes were directly or indirectly observed. However, this thicker barrier had a relative high rms roughness of *ca.* 0.6 nm. The high surface diffusion properties of graphene led to cluster-like Al_2_O_3_ film growth. This phenomenon limits the minimal possible thickness for complete barrier coverage on graphene surfaces using standard Al evaporation methods. The minimum required Al_2_O_3_ layer thickness for complete coverage of nominally 3 nm becomes *ca.* 5 nm in practice because of this clustering.

## Methods

Graphene flakes were obtained by micromechanical cleavage of single-crystal graphite^30^,^31^ (see Supplementary Fig. S1 online). The flakes were placed onto 90-nm-thick SiO_2_ films that had been thermally grown on silicon substrates. Monolayer graphenes were initially localized using optical microscopy. The crystalline quality and the number of layers of each flake were probed using Raman spectroscopy (Renishaw inVia confocal microscope operated with a 532-nm solid-state laser). Al_2_O_3_ barriers were then deposited by thermal evaporation of aluminum *in vacuo* (base pressure of 10^−7^ Torr) with posterior ambient oxidation. Different nominal Al_2_O_3_ barrier thicknesses (expansion factor of 1.28 from Al thickness) were evaluated; i.e., 1, 2 and 3 nm. The samples were then coated with a Co layer, which represented the ferromagnetic electrodes of a spintronic device. A 4-nm-thick Co layer was deposited by DC-magnetron sputtering at 3 mTorr of Ar, 130 mA (*ca.* 160 V) and a base pressure of 10^−7^ Torr. The samples were monitored by Raman spectroscopy between each fabrication step. A Cs-corrected FEI Titan 80/300 S/TEM microscope was used to obtain scanning and conventional transmission electron micrographs of the Co/Al_2_O_3_/graphene/SiO_2_ interfaces. High-angle annular dark field (HAADF) images were acquired using the STEM mode. Space-resolved elemental analyses were performed *via* energy dispersive X-ray (EDX) spectroscopy to map the layer interfaces. The chemical nature of the Al_2_O_3_ barriers was also analyzed using electron energy loss spectroscopy (EELS). The cross-sectional (S)-TEM samples were previously prepared using focused ion beam (FIB) protocols for lamella preparation (FEI Helios NanoLab DualBeam). Topographical aspects of the Al_2_O_3_ barriers were also acquired through atomic force microscopy measurements using a Bruker Multimode 8 AFM operated in the intermittent mode at a drive amplitude of 120 mV with a Si tip having k = 5 N/m.

## Additional Information

**How to cite this article**: Canto, B. *et al.* On the Structural and Chemical Characteristics of Co/Al_2_O_3_/graphene Interfaces for Graphene Spintronic Devices. *Sci. Rep.* **5**, 14332; doi: 10.1038/srep14332 (2015).

## Supplementary Material

Supplementary Information

## References

[b1] Tombros N., Jozsa C., Popinciuc M., Jonkman H. T. & van Wees B. J. Electronic spin transport and spin precession in single graphene layers at room temperature. Nature 448, 571–574 (2007). 17632544 10.1038/nature06037

[b2] Han W. *et al.* Electrical detection of spin precession in single layer graphene spin valves with transparent contacts. Appl. Phys. Lett. 94, 222109-1–222109-3 (2009).

[b3] Han W. *et al.* Tunneling spin injection into single layer graphene. Phys. Rev. Lett. 105, 167202-1–167202-4 (2010). 21231003 10.1103/PhysRevLett.105.167202

[b4] Han W. *et al.* R. K. Spin transport and relaxation in graphene. J. Magn. Magn. Mat. 324, 369–381 (2012).

[b5] Seneor P. *et al.* Spintronics with graphene. MRS Bulletin 37, 1245–1254 (2012).

[b6] Józsa C. *et al.* Linear scaling between momentum and spin scattering in graphene. Phys. Rev. B 80, 241403R (2009).

[b7] Popinciuc M. *et al.* Electronic spin transport in graphene field-effect transistors. Phys. Rev. B 80, 214427 (2009). 10.1103/PhysRevLett.100.236603 18643531

[b8] Schmidt G., Ferrand D., Molenkamp L. W., Filip A. T. & van Wees B. J. Fundamental obstacle for electrical spin injection from a ferromagnetic metal into a diffusive. Phys. Rev. B 62, R4790(R) (2000).

[b9] Rashba E. I. Theory of electrical spin injection: tunnel contacts as a solution of the conductivity mismatch problem. Phys. Rev. B 62, R16267(R) (2000).

[b10] Fert A. & Jaffre’s H. Conditions for efficient spin injection from a ferromagnetic metal into a semiconductor. Phys. Rev. B 64, 184420 (2001).

[b11] Maassen T., Vera-Marun I. J., Guimarães M. H. D. & van Wees B. J. Contact induced spin relaxation in hanle spin precession measurements. Phys. Rev. B 86, 235408 (2012).

[b12] Sosenko E., Wei H. & Vivek A. Effect of contacts on spin lifetime measurements in graphene. Phys. Rev. B 89, 245436 (2014).

[b13] Józsa C., Popinciuc M., Tombros N., Jonkman H. T. & van Wees B. J. Controlling the efficiency of spin injection into graphene by carrier drift. Phys. Rev. B 79, 081402R (2009).

[b14] Wang W. H. *et al.* Growth of atomically smooth MgO films on graphene by molecular beam epitaxy. Appl. Phys. Lett. 93, 183107 (2008).

[b15] Dlubak B. *et al.* Are Al_2_O_3_ and MgO Tunnel barriers suitable for spin injection in graphene? Appl. Phys. Lett. 97, 092502-1–092505-3 (2010).

[b16] Dlubak B. *et al.* Homogeneous pinhole free 1 nm Al_2_O_3_ tunnel barriers on graphene. Appl. Phys. Lett. 101, 203104-1–203104-3 (2012).

[b17] Robinson J. *et al.* Epitaxial graphene materials integration: effects of dielectric overlayers on structural and electronic properties. ACS Nano 25, 2667–72 (2010). 20415460 10.1021/nn1003138

[b18] Dlubak B., Kidambi P. R., Weatherup R. S., Hofmann S. & Robertson J. Substrate-assisted nucleation of ultra-thin dielectric layers on graphene by atomic layer deposition. Appl. Phys. Lett. 100, 173113 (2012).

[b19] Tombros N. *et al.* Anisotropic spin relaxation in graphene. Phys. Rev. Lett. 101, 046601 (2008). 18764351 10.1103/PhysRevLett.101.046601

[b20] Huertas-Hernando D., Guinea F. & Brataas A. Spin-orbit coupling in curved graphene, fullerenes, nanotubes, and nanotube caps. Phys. Rev. B 74, 155426 (2006).

[b21] Friedman A. L., van ’t Erve O. M. J., Li C. H., Robinson J. T. & Jonker B. T. Homoepitaxial tunnel barriers with functionalized graphene-on-graphene for charge and spin transport. Nat. Commun. 5, 3161 (2014). 24445349 10.1038/ncomms4161

[b22] Yamaguchi T. *et al.* Electrical spin injection into graphene through monolayer hexagonal boron nitride. Appl. Phys. Express 6, 073001 (2013).

[b23] Kamalakar M. V., Dankert A., Bergsten J., Ive T. & Dash S. P. Enhanced tunnel spin injection into graphene using chemical vapor deposited hexagonal boron nitride. Sci. Rep. 4, 6146 (2014). 25156685 10.1038/srep06146 PMC4143790

[b24] Friedman A. L., van ’t Erve O. M. J., Robinson J. T., Whitener K. E. & Jonker B. T. Hydrogenated graphene as a homoepitaxial tunnel barrier for spin and charge transport in graphene. ACS Nano 9, 6747 (2015). 26047069 10.1021/acsnano.5b02795

[b25] Binns C., Baker S. H., Demangeat C. & Parlebas J. C. Growth, electronic, magnetic and spectroscopic properties of transition metals on graphite surface. Science Reports 34, 105–170 (1999).

[b26] Feldhoff A., Pippel E. & Wolterdorf J. Interface engineering of carbon-fiber reinforced mg–al alloys. Adv. Eng. Mat. 2, 471 (2000).

[b27] Ahn C. C. & Krivanek O. L. EELS Atlas—A Reference Guide of Electron Energy Loss Spectra Covering All Stable Elements (Arizona State University HREM Facility & Gatan Inc. 1983).

[b28] Ferrari A. C. *et al.* Raman spectrum of graphene and graphene layers. Phys. Rev. Lett. 97, 187401-1–187401-4 (2006). 17155573 10.1103/PhysRevLett.97.187401

[b29] Ziegler J. F., Ziegler M. D. & Biersack J. P. SRIM - The stopping and range of ions in matter. Nucl. Instrum. Methods B 268, 1818–1823 (2010).

[b30] Novoselov K. S. *et al.* Electric field effect in atomically thin carbon films. Science 306, 666–669 (2004). 15499015 10.1126/science.1102896

[b31] Klar P. *et al.* Raman scattering efficiency of graphene. Phys. Rev. B 87, 205435-1–205435-12 (2013).

