# Supplementary material for: On the Structural and Chemical Characteristics of Co/Al2O3/graphene Interfaces for Graphene Spintronic Devices
Source: Sci Rep. 2015 Sep 23;5:14332. doi: 10.1038/srep14332 (PMC4585803; doi:10.1038/srep14332)
Supplement: Supplementary Information [file srep14332-s1.pdf]

**Supplementary Information**

# On the Structural and Chemical Characteristics of $Co/Al_2O_3$/graphene Interfaces for Graphene Spintronic Devices

Barbara Canto[1], Cristol P. Gouvea[2], Braulio S. Archanjo[2], João E. Schmidt[1] & Daniel L. Baptista[1,*]

[1]Instituto de Física, Universidade Federal do Rio Grande do Sul, Porto Alegre, 91501-970, Brazil.

[2]Divisão de Metrologia de Materiais, INMETRO, Duque de Caxias, 25250-020, Brazil.

*Corresponding author: dbaptista@gmail.com

SI-1 Micromechanical Cleavage and Optical Micrographs

Graphene flakes were obtained by micromechanical cleavage of single-crystal graphite (Nacional de Grafite LTDA) using Nitto tape. The flakes were placed onto 90-nm-thick $SiO_2$ films that had been thermally grown on silicon substrates. Monolayer graphenes were initially localized using optical microscopy (Figure S1).

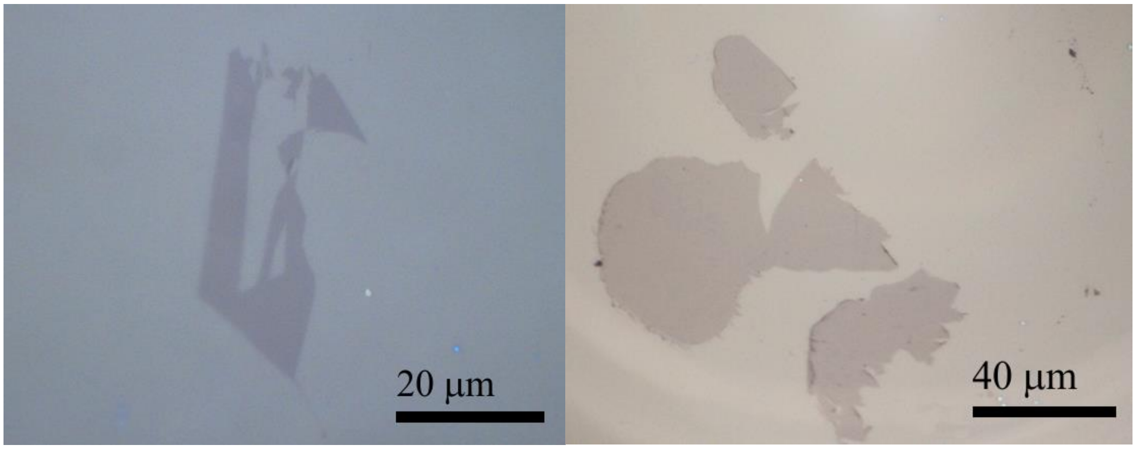


Figure S1. Optical micrographs of monolayer graphene flakes placed onto 90-nm-thick $SiO_2$ films.

SI-2 Atomic Force Microscopy Analysis

$Al_2O_3$ barriers were deposited onto the graphene surface by thermal evaporation of aluminum *in vacuo* (base pressure of $10^{-7}$ Torr) with posterior ambient oxidation. Figure S2 shows topographical AFM images of samples with nominally 1- and 3-nm-thick $Al_2O_3$ barriers. The 1-nm-thick barrier presents an incomplete surface coverage as consequence of a cluster-like film (Volmer-Weber) growth. This clustering phenomenon limits the minimal thickness possible for complete barrier coverage on graphene surfaces using standard Al evaporation methods. The minimum required $Al_2O_3$ layer thickness for complete coverage of nominally 3 nm becomes *ca.* 5 nm in practice because of this clustering. Although the nominal (3 nm) thickness may lead to complete barrier coverage a relative high rms roughness of ~0.6 nm is observed.

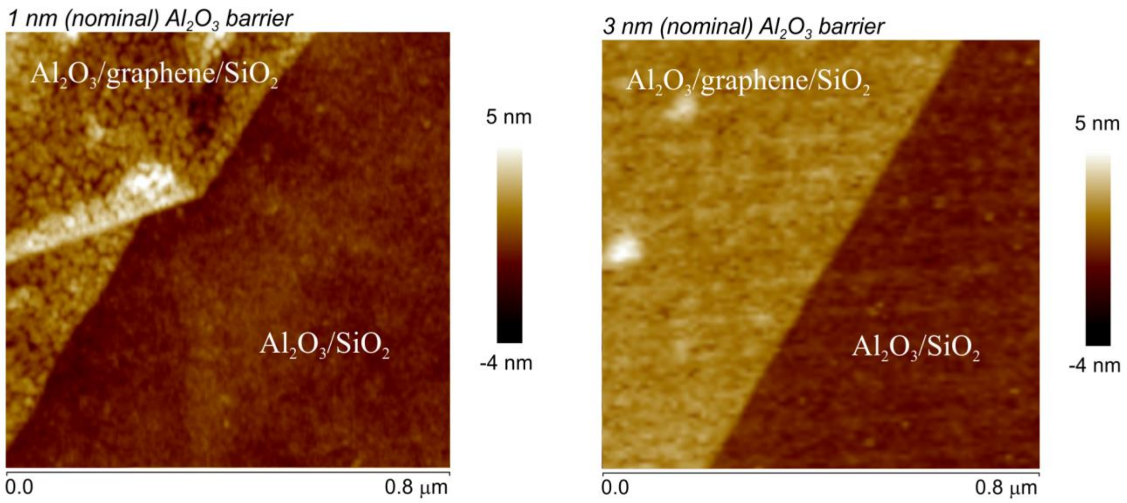


Figure S2. Topographical AFM images of samples with nominally 1- and 3-nm-thick $Al_2O_3$ barriers.

## SI-3 STEM-HAADF Image of Many Pinhole Cobalt Contacts

Figure S3 shows a cross-sectional HAADF-STEM image of monolayer graphene sample with nominally 1-nm-thick $Al_2O_3$ barrier. It clearly indicates the presence of inhomogeneous barrier containing many pinholes. The Co layer contacts the graphene directly in those pinhole regions.

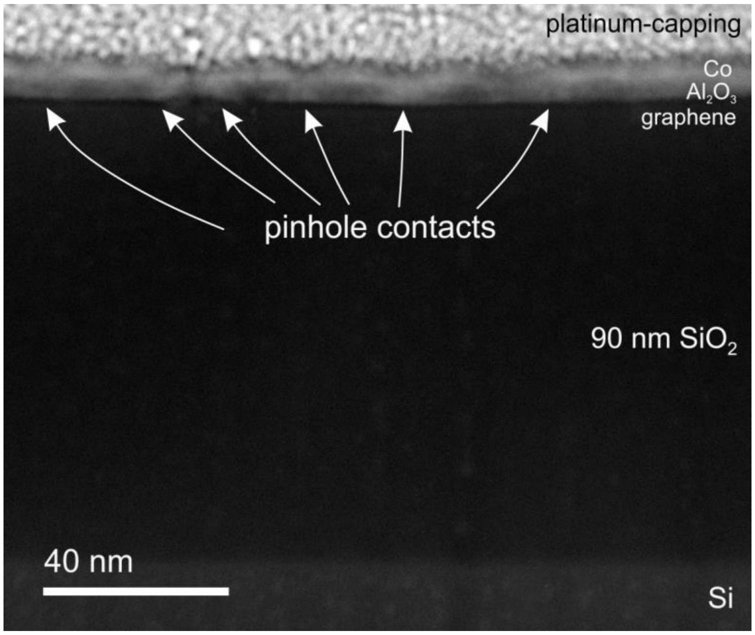


Figure S3. HAADF-STEM cross-sectional full-image of monolayer graphene sample with nominally 1-nm-thick $Al_2O_3$ barrier.
